# Correction: Sadeghi et al. lncRNA–miRNA–mRNA ceRNA Network Involved in Sheep Prolificacy: An Integrated Approach. *Genes* 2022, *13*, 1295

**DOI:** 10.3390/genes16030299

**Published:** 2025-02-28

**Authors:** Masoumeh Sadeghi, Abolfazl Bahrami, Aliakbar Hasankhani, Hamed Kioumarsi, Reza Nouralizadeh, Sarah Ali Abdulkareem, Farzad Ghafouri, Herman W. Barkema

**Affiliations:** 1 Environmental Health, Zahedan University of Medical Sciences, Zahedan 98, Iran; sadeghimasoome2@gmail.com; 2 Department of Animal Science, College of Agriculture and Natural Resources, University of Tehran, Karaj 31, Iran; a.hasankhani74@ut.ac.ir (A.H.); farzad.ghafouri2@ut.ac.ir (F.G.); 3 Department of Animal Science Research, Gilan Agricultural and Natural Resources Research and Education Center, Agricultural Research, Education and Extension Organization (AREEO), Rasht 43, Iran; hkioumarsi@yahoo.com; 4 Department of Food and Drug Control, Faculty of Pharmacy, Jundishapour University of Medical Sciences, Ahvaz 63, Iran; 5 Department of Computer Science, Al-Turath University College, Al Mansour, Baghdad 10011, Iraq; sarah.ali@turath.edu.iq; 6 Department of Production Animal Health, Faculty of Veterinary Medicine, University of Calgary, Calgary, AB T2N 4Z6, Canada; barkema@ucalgary.ca

In the original publication [1], there was an error regarding the third affiliation for Abolfazl Bahrami. With this correction, the original third affiliation has been removed, and the order of the rest affiliations have been adjusted accordingly.

The authors state that the scientific conclusions are unaffected. This correction was approved by the Academic Editor. The original publication has also been updated.
