# Supplementary material for: lncRNA–miRNA–mRNA ceRNA Network Involved in Sheep Prolificacy: An Integrated Approach
Source: Genes (Basel). 2022 Jul 22;13(8):1295. doi: 10.3390/genes13081295 (PMC9332185; doi:10.3390/genes13081295)

Supplementary Figure S1: The result of reads correspondent to the AGR2 gene for validation of alignment related to transcripts.

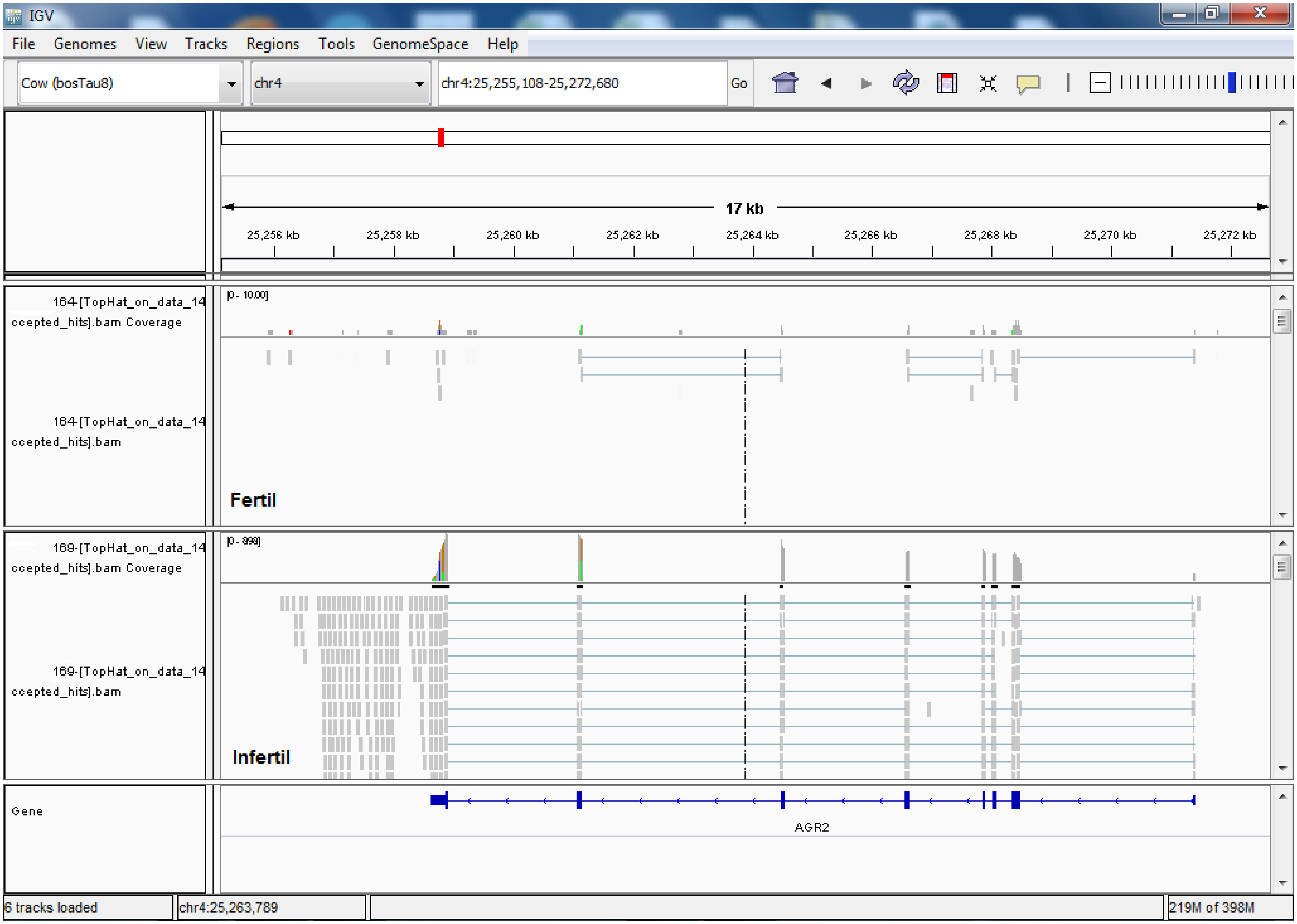

Supplement: Supplementary file 1 [file genes-13-01295-s001.zip › Figure S1.pdf]
